# Crystal structure and Hirshfeld surface analysis of (nitrato-κ^2^*O*,*O*′)(1,4,7,10-tetra­aza­cyclo­dodecane-κ^4^*N*)nickel(II) nitrate

**DOI:** 10.1107/S2056989024009496

**Published:** 2024-10-11

**Authors:** Joseph Reibenspies, Nadia Small, Nattamai Bhuvanesh, Gina Chiarella, Vivian Salazar, Bréayshia Pery, Rukiyah Smith, Deja Toole, Shamika Hewage, Harschica Fernando, Eric Reinheimer

**Affiliations:** a Department of Chemistry, Texas A&M University, College Station, Texas 77843, USA; b Prairie View A&M University,Prairie View Texas 77446 USA; c Rigaku Americas Corporation, The Woodlands Texas 77381 USA; University of Kentucky, USA

**Keywords:** crystal structure, cyclen, nickel, nitrate

## Abstract

The crystal structure of the title compound, [Ni(C_8_H_20_N_4_)(NO_3_)]NO_3_, at room temperature, has monoclinic (*P*2_1_/*n*) symmetry. The structure displays inter­molecular hydrogen bonding. The nickel displays a distorted bipyramidal geometry with the symmetric bidentate bonded nitrate occupying an equatorial site. The 1,4,7,10-tetra­aza­cyclo­dodecane (cyclen) backbone has the [4,8] configuration, with three nitro­gen-bound H atoms directed above the plane of the nitro­gen atoms towards the offset nickel atom with the fourth nitro­gen-bound hydrogen directed below from the plane of the nitro­gen atoms. The nitrate anion O atoms are seen to hydrogen bond to the H atoms bound to the N atoms of the ligand.

## Abstract 2

The crystal structure of [(1,4,7,10-tetra­aza­cyclo­dodeca­ne)(nitrate)]nickel(II) nitrate, at room temperature, has monoclinic (*P*2_1_/*n*) symmetry. The structure displays inter­molecular hydrogen bonding.

## 1. Chemical context

The scientific community has long been acquainted with 1,4,7,10-tetra­aza­cyclo­dodecane (cyclen) transition-metal complexes to nitrate with nitrate as a counter-ion. Nevertheless, it is important to emphasize that the structure of the coordinated complex of nickel(cyclen) and nitrate, with nitrate as the counter-ion, has not been previously documented. The absence of such a publication can be attributed to the difficulties encountered in crystallizing the [Ni(cyclen)NO_3_]NO_3_ complex. This manuscript provides a comprehensive overview of the synthesis, crystallization, and structural analysis of [Ni(cyclen)NO_3_]NO_3_. It is noteworthy to mention that this manuscript represents the culmination of a crystallographic workshop conducted by students and faculty of Prairie View A&M and Texas A&M Universities.
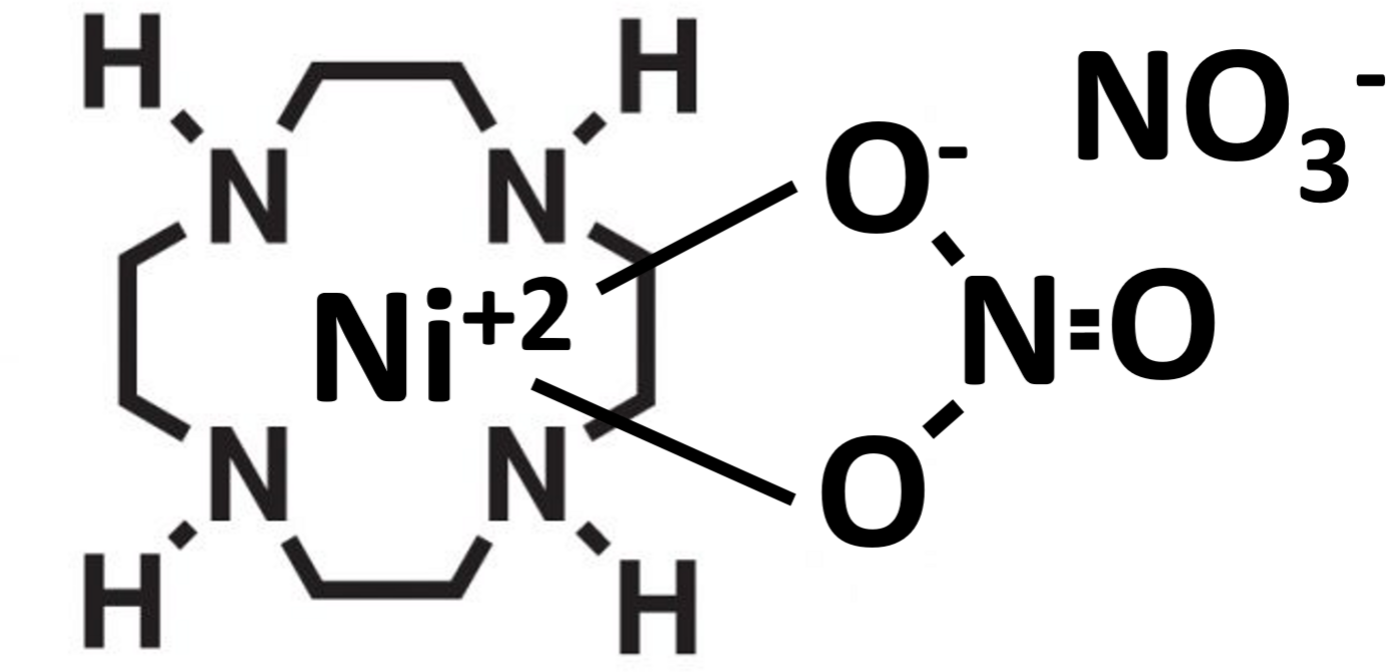


## 2. Structural commentary

**Figure 1:**
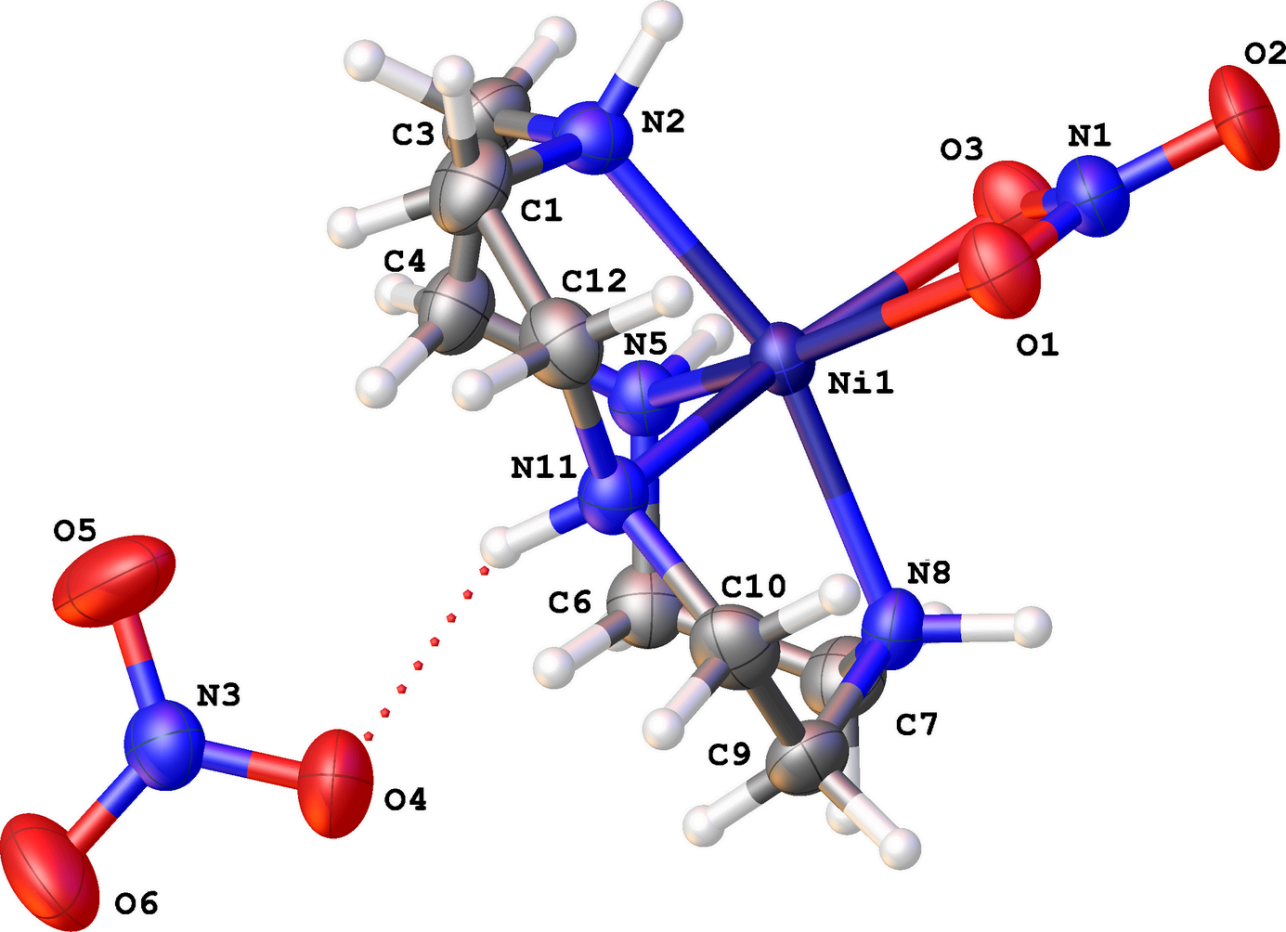
Displacement plot (50% probability ellipsoids) of [Ni(cyclen)NO_3_]NO_3._

Fig. 1 ▸ illustrates the structure of the title compound, which crystallizes in the *P*2_1_/*n* space group and also displays a cyclen backbone that exhibits the [4,8] configuration (four bonds between the corner atoms C3 and C7 and eight bonds between C7 back to C3; Meyer *et al.*, 1998 ▸). Three of the four hydrogen atoms bound to nitro­gens (N2, N5, N8) of the cyclen ring are positioned above the plane of the nitro­gen atoms and on the same side as the displaced nickel atom, and the hydrogen bound to the remaining nitro­gen (N11) is below the plane of the nitro­gen atoms. The nickel atom can be characterized as having a distorted trigonal–bipyramidal structure, with N2 and N8 (cyclen) occupying the axial positions, and N5 (cyclen), N11 (cyclen), and the bidentate nitrate occupying the equatorial sites. There are two nitrates present, one nitrate is the counter-ion and the second nitrate is bonded to the nickel in a bidentate B01 configuration (Morozov *et al.*, 2008 ▸). The cause of distortion to the expected [3,3,3,3] conformation (as seen in Cu^II^ structures) is unknown but can possibly be attributed to the Jahn–Teller effect expected in the Cu^II^ species *versus* Ni^II^, which is greater for Cu^II^ than for Ni^II^ (Reinen *et al.*, 1988 ▸).

There exists a ‘twisting’ around the trigonal plane (of the trigonal–bipyramidal structure) of nitrate oxygens to the nitro­gen N11 – H (on N11) to N5 – H (on N5). In the Cu^II^ cyclen nitrate study by Gasser *et al.* (2007 ▸), they showed that there was repulsion between the hydrogen of the nitro­gen between the two most distorted angles, which caused a longer Cu—O bond on that side of the complex *versus* the Cu—O bond closer to the more symmetric N—Cu—N angles. This distortion, potentially caused by the nitrate, is also seen in the nickel macrocycle reported here, to the point of causing a shift in the coordination sphere geometry of the nickel.

Additionally, the nitrate group bond in [Ni(cyclen)NO_3_]NO_3_, has a distorted bidentate bond to the nickel atom [Ni1⋯O1 = 2.151 (3) Å, Ni1⋯ O3 2.113 (3) Å] with a potentially large area of inter­action with nickel’s coordination space. The nitrate ligand is bound by two separate bonds (bidentate) and occupies more than a single bond space, with its resonance causing an increase of ligand energy close to the metal. This contributes to the ‘twisting’ of the structure that was also seen in the similar structure of nickel cyclen acetate (Verma *et al.*, 2022 ▸). Most notably, copper and nickel structures of the same form, experience distortions to their cyclen conformations when nitrate is present. For copper, there is a distortion to the [3,3,3,3] symmetry and for nickel, there is a ‘twisting’ causing a [4,8] conformation.

## 3. Supra­molecular features

**Figure 2:**
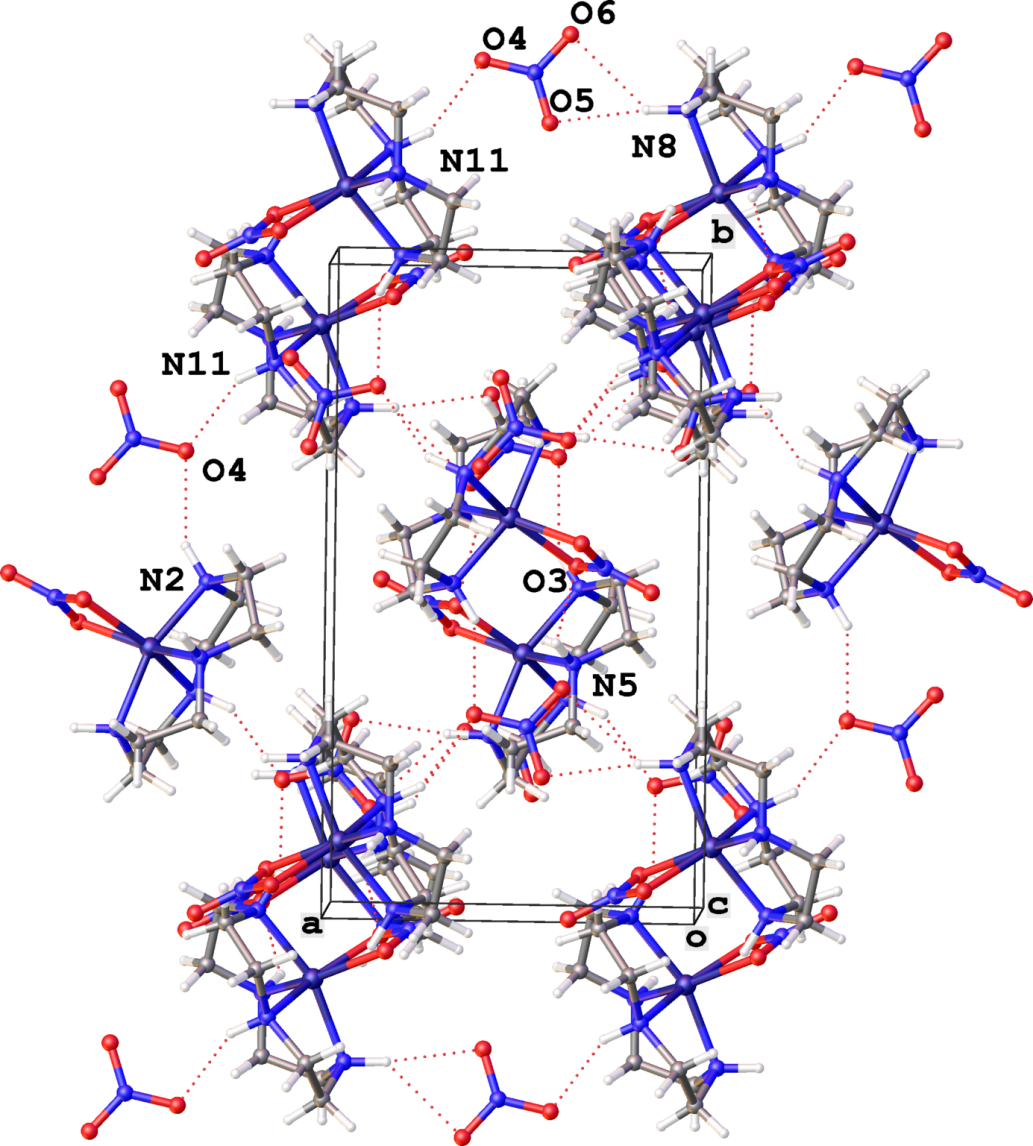
A packing plot (with unit-cell box, viewed down the *c* axis, where the *a* axis is horizontal and the *b* axis is vertical) highlighting the hydrogen bonds to NO_3_^−^ in [Ni(cyclen)NO_3_]NO_3_. Dashed lines indicate hydrogen bonds (Table 1 ▸).

The anion, NO_3_^−^, forms a hydrogen bond with the hydrogen bound to N2 of the cyclen ring and the hydrogen bound to N11 of an adjacent [Ni(cyclen)NO_3_]^+^ complex, effectively bridging the two cationic complexes through the same nitrate oxygen atom (O4) (see Fig. 2 ▸, Table 1 ▸). The remaining oxygens atoms of the anion (O5, O6) form hydrogen bonds to the hydrogen bound to N8 of an adjacent cation. Hydrogen bonds to the NO_3_^−^ anion are: N11⋯O4 = 3.030 (5) Å, N2⋯O4(−*x* + 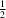, *y* + 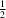, −*z* + 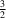) = 3.091 (5) Å, N8⋯O5(*x* + 1, *y*, *z*) = 3.253 (5) Å, N8 ⋯ O6(*x* + 1, *y*, *z*) = 3.273 (5) Å and to the bound nitrate the distances are: N5⋯O3(−*x* + 1, −*y* + 1, −*z* + 1) = 3.130 (4) Å, see Fig. 2 ▸.

**Figure 3:**
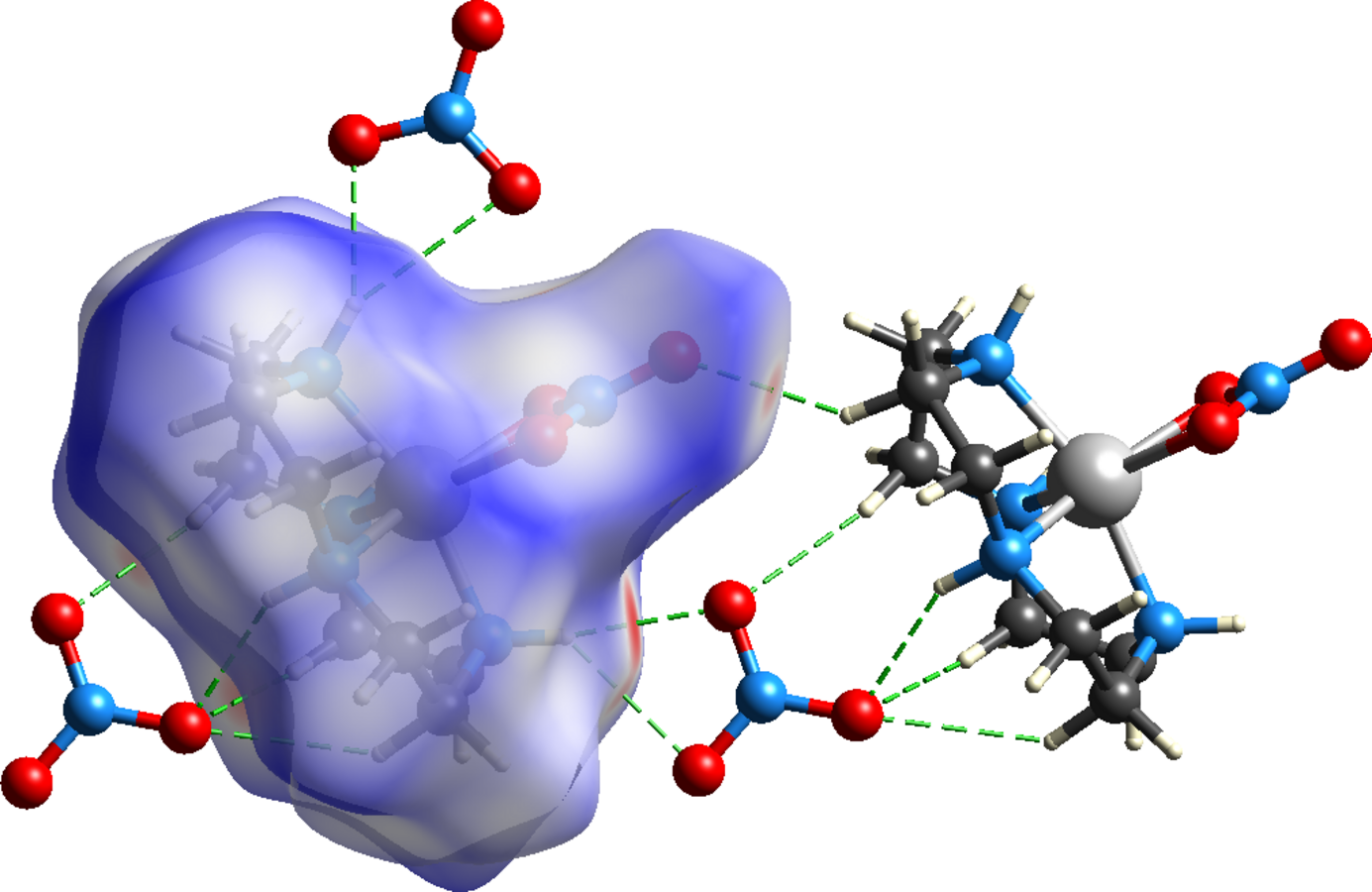
Hirshfeld surface plot of [Ni(cyclen)NO_3_]NO_3_. Dashed lines indicate hydrogen bonds and close contacts.

**Figure 4:**
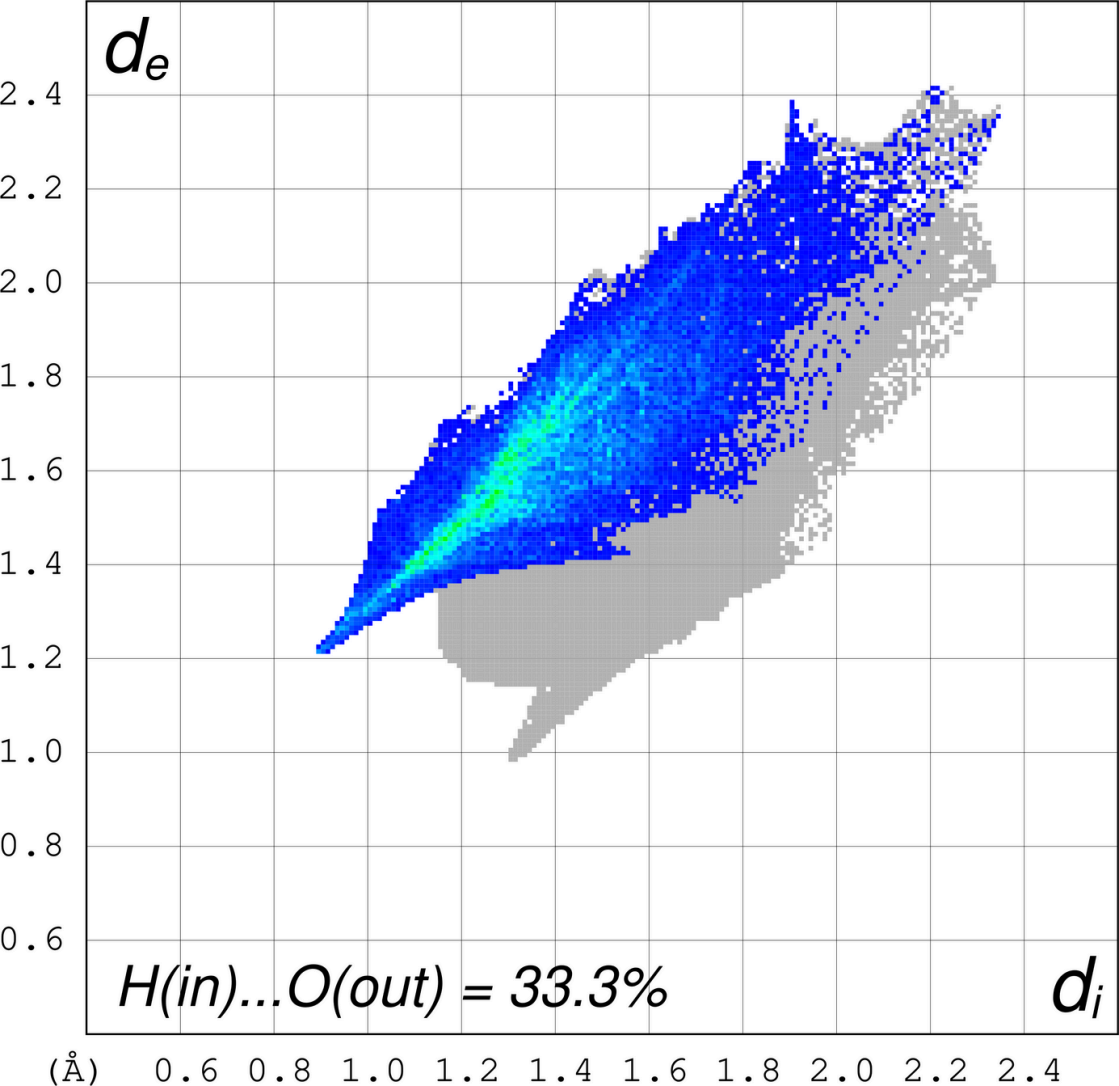
Fingerprint plot of the close contacts between [Ni(cyclen)NO_3_]^+^ cation H atoms to the adjacent nitrate anion O atoms [H(in)⋯O(out)], which equals 33.3% of the surface area.

**Figure 5:**
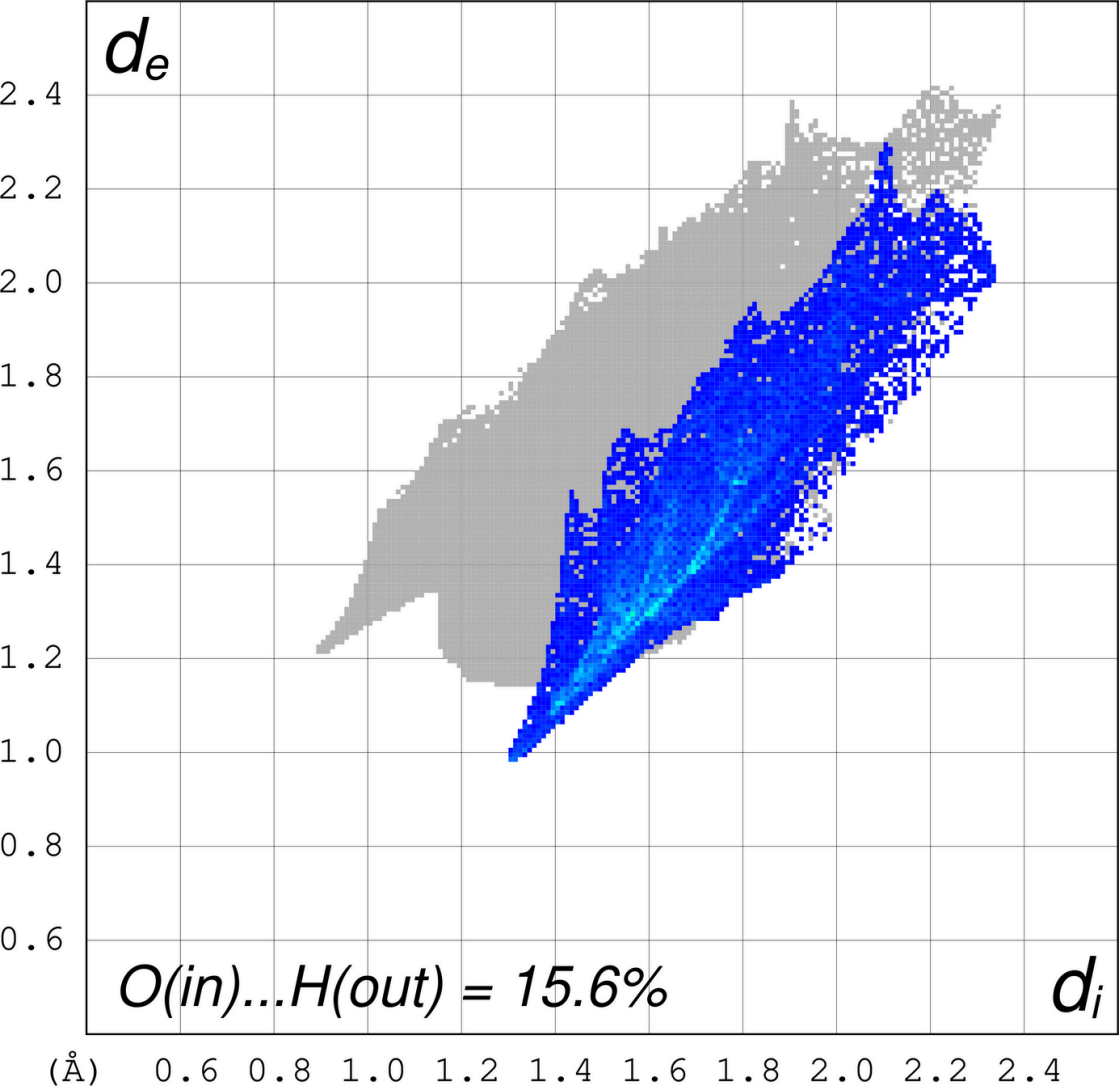
Fingerprint plot of the close contacts between [Ni(cyclen)NO_3_]^+^ cation O atoms to adjacent [Ni(cycle)NO_3_] H atoms [O(in)⋯H(out)], which equals 15.6% of the surface area.

Fig. 3 ▸ shows the Hirshfeld surface (Spackman *et al.*, 2021 ▸) and indicates short contacts for hydrogens bound to nitro­gen and nitrate oxygens of the unbound nitrate (anion) and a slightly longer contact between the carbon hydrogens and the terminal oxygen of the bound nitrate, while the bound oxygen atom of the bound nitrate forms a long hydrogen bond to the nitro­gen atom of a nearby cation. The fingerprint plot (Fig. 4 ▸) indicates that the [Ni(cyclen)NO_3_]^+^ hydrogens to nitrate anion oxygen contacts make about 33.3% (N—H_inside_⋯O_outside_) of all close contacts, while the fingerprint plot (Fig. 5 ▸) indicates that the [Ni(cyclen)NO_3_]^+^ oxygens to [Ni(cyclen)NO_3_]^+^ hydrogens (adjacent mol­ecules) make about 15.6% [O_inside_⋯H—N_outside_ and O_inside_⋯H—C_outside_] of the close contacts.

## 4. Database survey

A search of the Cambridge Structural Database (CSD, updated to June 2024, Groom *et al.*, 2016 ▸) found the [Ni(cyclen)acetate]Br complex (CSD refcode: KALQUN; Verma *et al.*, 2022 ▸), which displays a similar trigonal–bipyramidal nickel complexed to a cyclen in a [4,8] configuration with a bidentate acetate bound in one of the axial positions of the trigonal bipyramid. This configuration is relatively common for nickel cyclen complexes as opposed to the similar size copper cyclen complexes, which have the [3,3,3,3] ‘square’ configuration (Verma *et al.*, 2022 ▸). However, Gasser *et al.* (2007 ▸) reported a distortion to some copper cyclen complex geometries including one with a monodentate nitrate (CSD refcode: TZCDCU; Clay *et al.*, 1979 ▸) and one with an additional ligand (ferrocene meth­yl) that caused the nitrate to appear bidentate (CSD refcode: UDINOL; Gasser *et al.* 2007 ▸). They suspect that the second bond of the nitrate to the copper was electrostatic due to the steric inter­ference of the ligand on the cyclen. The zinc cyclen nitrate compound, as described by Vargova *et al.* (2007 ▸) (CSD refcode: MIKBOY), displays the nitrate ligand in a monodentate coordination, while the tetra­methyl­cyclen nickel nitrate structure, as reported by Yenuganti *et al.* (2020 ▸) (CSD refcode: XACDEO), showcases a symmetrical bidentate B01 nitrate ligand. Furthermore, both structures share the cyclen backbone in the [3,3,3,3] ‘square’ structure, which is also seen in the uncomplexed (free) cyclen ligand (CSD refcode:VUCGEF; Reibenspies, 1992 ▸)

## 5. Synthesis and crystallization

0.2 g of cyclen were added to a solution of Ni(NO_3_)_2_·6H_2_O (0.3 g dissolved in 1 ml of distilled water), resulting in the formation of a deep-blue solution, which was then transferred to a 5 ml uncapped vial, which was placed inside a 10 ml vial. The 10 ml vial was filled with 3 ml of absolute ethanol (outside of the 5 ml vial). The 10 ml vial was capped and after 24 h, the ethanol had diffused into the aqueous solution, but no crystals were observed. To address this, the cap of the vapor diffusion apparatus (10 ml vial) was removed, allowing the aqueous/ethanol solution to evaporate. After an additional 24 h, light-blue crystals were discovered above the concentrated blue solution and collected from the 5 ml vial. It is important to note that normal evaporation of the aqueous solution will yield a blue oil without any crystallization.

## 6. Refinement details

Crystal data, data collection and structure refinement details are summarized in Table 2 ▸. During the final stages of refinement a twin was detected from analysis of the structure factor file (FCF), which contains the calculated and observed structure factors (Dolomanov *et al.*, 2009 ▸). The refinement of the twin (1 0 0.139 0 − 1 0 0 0 − 1, BASF of 0.177, twofold about the *a* axis) resulted in an improved structure and a decrease in the residual values. Publication documents were generated with the program *publCIF* (Westrip, 2010 ▸).

## Supplementary Material

Crystal structure: contains datablock(s) global, I. DOI: 10.1107/S2056989024009496/pk2709sup1.cif

Structure factors: contains datablock(s) I. DOI: 10.1107/S2056989024009496/pk2709Isup3.hkl

CCDC reference: 2386999

Additional supporting information:  crystallographic information; 3D view; checkCIF report

## Figures and Tables

**Table 1:** Hydrogen-bond geometry (Å, °)

*D*—H⋯*A*	*D*—H	H⋯*A*	*D*⋯*A*	*D*—H⋯*A*
N2—H2⋯O4^i^	0.98	2.22	3.091 (5)	147
N5—H5⋯O3^ii^	0.98	2.32	3.130 (4)	139
N8—H8⋯O5^iii^	0.98	2.28	3.253 (5)	172
N8—H8⋯O6^iii^	0.98	2.50	3.273 (5)	135
N11—H11⋯O4	0.98	2.14	3.030 (5)	151

Symmetry codes: (i) 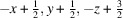; (ii) 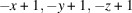; (iii) 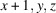.

**Table 2:** Experimental details

Crystal data	
Chemical formula	[Ni(C_8_H_20_N_4_)(NO_3_)]NO_3_
*M*_r_	355.01
Crystal system, space group	Monoclinic, *P*2_1_/*n*
Temperature (K)	293
*a*, *b*, *c* (Å)	8.7321 (5), 15.2444 (9), 10.8545 (6)
β (°)	94.973 (5)
*V* (Å^3^)	1439.47 (14)
*Z*	4
Radiation type	Mo *K*α
μ (mm^−1^)	1.39
Crystal size (mm)	0.4 × 0.3 × 0.02

Data collection	
Diffractometer	XtaLAB Mini II
Absorption correction	Multi-scan (*CrysAlis PRO*; Rigaku OD, 2024 ▸)
*T*_min_, *T*_max_	0.899, 1.000
No. of measured, independent and observed [*I* > 2σ(*I*)] reflections	12195, 12195, 9604
*R*_int_	0.038

Refinement	
*R*[*F*^2^ > 2σ(*F*^2^)], *wR*(*F*^2^), *S*	0.036, 0.104, 1.09
No. of reflections	12195
No. of parameters	191
H-atom treatment	H-atom parameters constrained
Δρ_max_, Δρ_min_ (e Å^−3^)	0.39, −0.28

Computer programs: *CrysAlis PRO* (Rigaku OD, 2024 ▸), *SHELXT* (Sheldrick, 2015*a* ▸), *SHELXL2019/2* (Sheldrick, 2015*b* ▸) and *OLEX2* (Dolomanov *et al.*, 2009 ▸).

## supplementary crystallographic information

### (Nitrato-κ2O,O')(1,4,7,10-tetraazacyclododecane-κ4N)nickel(II) nitrate Crystal data

**Table:**

[Ni(C_8_H_20_N_4_)(NO_3_)]NO_3_	*F*(000) = 744
*M**_r_* = 355.01	*D*_x_ = 1.638 Mg m^−^^3^
Monoclinic, *P*2_1_/*n*	Mo *K*α radiation, λ = 0.71073 Å
*a* = 8.7321 (5) Å	Cell parameters from 4001 reflections
*b* = 15.2444 (9) Å	θ = 2.3–29.9°
*c* = 10.8545 (6) Å	µ = 1.39 mm^−^^1^
β = 94.973 (5)°	*T* = 293 K
*V* = 1439.47 (14) Å^3^	Plate, blue
*Z* = 4	0.4 × 0.3 × 0.02 mm

### (Nitrato-κ2O,O')(1,4,7,10-tetraazacyclododecane-κ4N)nickel(II) nitrate Data collection

**Table:**

XtaLAB Mini II diffractometer	9604 reflections with *I* > 2σ(*I*)
Detector resolution: 10.0000 pixels mm^-1^	*R*_int_ = 0.038
ω scans	θ_max_ = 26.4°, θ_min_ = 2.3°
Absorption correction: multi-scan (CrysAlisPro; Rigaku OD, 2024)	*h* = −10→8
*T*_min_ = 0.899, *T*_max_ = 1.000	*k* = −19→19
12195 measured reflections	*l* = −13→13
12195 independent reflections	

### (Nitrato-κ2O,O')(1,4,7,10-tetraazacyclododecane-κ4N)nickel(II) nitrate Refinement

**Table:**

Refinement on *F*^2^	Primary atom site location: dual
Least-squares matrix: full	Hydrogen site location: inferred from neighbouring sites
*R*[*F*^2^ > 2σ(*F*^2^)] = 0.036	H-atom parameters constrained
*wR*(*F*^2^) = 0.104	*w* = 1/[σ^2^(*F*_o_^2^) + (0.0406*P*)^2^ + 0.8788*P*] where *P* = (*F*_o_^2^ + 2*F*_c_^2^)/3
*S* = 1.09	(Δ/σ)_max_ < 0.001
12195 reflections	Δρ_max_ = 0.39 e Å^−^^3^
191 parameters	Δρ_min_ = −0.28 e Å^−^^3^
0 restraints	

### (Nitrato-κ2O,O')(1,4,7,10-tetraazacyclododecane-κ4N)nickel(II) nitrate Special details

**Table:**

Experimental. Single crystals of C_8_H_20_N_6_NiO_6_ [Ni(cyclen)NO_3_]NO_3_ were obtained by vapor diffusion followed by evaporation. A suitable crystal was selected and mounted on a RIGAKU XtaLAB Mini II diffractometer. The crystal was kept at 293 (2) K during data collection (*CrysAlis PRO* : Rigaku Oxford Diffraction, 2024). Employing *Olex2* (Dolomanov *et al.*, 2009), the structure was solved with the *SHELXT* (Sheldrick, 2015a) structure solution program and refined with the *SHELXL* (Sheldrick, 2015b) refinement package using full-matrix least-squares minimization.
Geometry. All esds (except the esd in the dihedral angle between two l.s. planes) are estimated using the full covariance matrix. The cell esds are taken into account individually in the estimation of esds in distances, angles and torsion angles; correlations between esds in cell parameters are only used when they are defined by crystal symmetry. An approximate (isotropic) treatment of cell esds is used for estimating esds involving l.s. planes.
Refinement. Refined as a 2-component twin.

### (Nitrato-κ2O,O')(1,4,7,10-tetraazacyclododecane-κ4N)nickel(II) nitrate Fractional atomic coordinates and isotropic or equivalent isotropic displacement parameters (Å^2^)

**Table:**

	*x*	*y*	*z*	*U*_iso_*/*U*_eq_	
Ni1	0.47429 (5)	0.40178 (3)	0.71868 (4)	0.02627 (16)	
O1	0.6641 (3)	0.44290 (18)	0.8450 (2)	0.0402 (7)	
O2	0.8551 (3)	0.5139 (2)	0.7739 (3)	0.0626 (10)	
O3	0.6572 (3)	0.47217 (18)	0.6500 (2)	0.0414 (8)	
N1	0.7303 (4)	0.4775 (2)	0.7575 (3)	0.0395 (9)	
N2	0.3234 (4)	0.5046 (2)	0.7480 (3)	0.0323 (8)	
H2	0.381537	0.558870	0.764991	0.039*	
N5	0.3440 (3)	0.38312 (19)	0.5547 (3)	0.0292 (8)	
H5	0.392730	0.413234	0.488510	0.035*	
N8	0.5615 (3)	0.2762 (2)	0.6909 (3)	0.0313 (8)	
H8	0.674094	0.277112	0.700910	0.038*	
N11	0.3508 (3)	0.3385 (2)	0.8461 (3)	0.0298 (8)	
H11	0.255201	0.314213	0.805949	0.036*	
C1	0.2362 (5)	0.4818 (3)	0.8563 (4)	0.0418 (11)	
H1A	0.133206	0.462931	0.827328	0.050*	
H1B	0.227560	0.533312	0.907739	0.050*	
C3	0.2243 (5)	0.5135 (3)	0.6305 (4)	0.0396 (11)	
H3A	0.274368	0.551436	0.574398	0.048*	
H3B	0.127546	0.540508	0.646862	0.048*	
C4	0.1937 (4)	0.4249 (3)	0.5703 (4)	0.0362 (10)	
H4A	0.134359	0.388525	0.621993	0.043*	
H4B	0.135815	0.431978	0.490575	0.043*	
C6	0.3441 (4)	0.2873 (3)	0.5311 (3)	0.0344 (10)	
H6A	0.310735	0.275553	0.445087	0.041*	
H6B	0.274129	0.258079	0.582361	0.041*	
C7	0.5067 (5)	0.2534 (3)	0.5614 (4)	0.0386 (11)	
H7A	0.508492	0.190241	0.550862	0.046*	
H7B	0.574574	0.279217	0.505188	0.046*	
C9	0.5013 (5)	0.2164 (3)	0.7832 (4)	0.0379 (11)	
H9A	0.579747	0.173954	0.810634	0.045*	
H9B	0.413183	0.184602	0.745263	0.045*	
C10	0.4544 (5)	0.2678 (3)	0.8934 (4)	0.0399 (11)	
H10A	0.402253	0.229681	0.947894	0.048*	
H10B	0.544393	0.292262	0.939630	0.048*	
C12	0.3177 (5)	0.4090 (3)	0.9320 (3)	0.0382 (10)	
H12A	0.412467	0.430823	0.974336	0.046*	
H12B	0.252712	0.387293	0.993362	0.046*	
O4	0.1146 (4)	0.2042 (2)	0.7487 (3)	0.0663 (10)	
O5	−0.0669 (4)	0.2984 (2)	0.7302 (4)	0.0846 (13)	
O6	−0.1194 (4)	0.1630 (2)	0.7286 (4)	0.0803 (12)	
N3	−0.0252 (4)	0.2221 (3)	0.7343 (3)	0.0398 (9)	

### (Nitrato-κ2O,O')(1,4,7,10-tetraazacyclododecane-κ4N)nickel(II) nitrate Atomic displacement parameters (Å^2^)

**Table:**

	*U*^11^	*U*^22^	*U*^33^	*U*^12^	*U*^13^	*U*^23^
Ni1	0.0229 (3)	0.0294 (3)	0.0263 (3)	−0.0023 (3)	0.0015 (2)	0.0014 (3)
O1	0.0360 (17)	0.050 (2)	0.0343 (16)	−0.0097 (15)	0.0008 (13)	0.0018 (14)
O2	0.0263 (17)	0.059 (2)	0.101 (3)	−0.0173 (17)	0.0009 (18)	0.000 (2)
O3	0.0414 (17)	0.048 (2)	0.0355 (16)	−0.0093 (16)	0.0057 (14)	0.0057 (15)
N1	0.029 (2)	0.036 (2)	0.053 (2)	−0.0034 (18)	0.0035 (18)	−0.0012 (19)
N2	0.0348 (19)	0.0290 (19)	0.0328 (18)	−0.0017 (17)	0.0005 (15)	−0.0021 (16)
N5	0.0276 (18)	0.033 (2)	0.0273 (17)	−0.0006 (16)	0.0020 (14)	0.0025 (15)
N8	0.0221 (17)	0.036 (2)	0.0353 (19)	0.0023 (16)	0.0023 (15)	0.0006 (16)
N11	0.0271 (18)	0.034 (2)	0.0284 (18)	0.0007 (16)	0.0028 (14)	0.0029 (16)
C1	0.038 (3)	0.050 (3)	0.037 (2)	0.009 (2)	0.007 (2)	−0.008 (2)
C3	0.041 (3)	0.037 (3)	0.040 (2)	0.010 (2)	−0.001 (2)	0.004 (2)
C4	0.031 (2)	0.045 (3)	0.032 (2)	0.006 (2)	−0.0025 (18)	−0.002 (2)
C6	0.035 (2)	0.041 (3)	0.028 (2)	−0.001 (2)	0.0012 (18)	−0.006 (2)
C7	0.041 (3)	0.038 (3)	0.038 (2)	0.004 (2)	0.007 (2)	−0.006 (2)
C9	0.037 (3)	0.031 (2)	0.045 (2)	0.006 (2)	0.001 (2)	0.006 (2)
C10	0.044 (3)	0.040 (3)	0.036 (2)	−0.003 (2)	0.003 (2)	0.011 (2)
C12	0.039 (2)	0.045 (3)	0.031 (2)	−0.004 (2)	0.0088 (19)	0.000 (2)
O4	0.0309 (18)	0.064 (2)	0.105 (3)	0.0029 (18)	0.0066 (19)	0.005 (2)
O5	0.087 (3)	0.045 (2)	0.128 (4)	0.025 (2)	0.045 (3)	0.020 (2)
O6	0.054 (2)	0.075 (3)	0.113 (3)	−0.029 (2)	0.012 (2)	−0.026 (2)
N3	0.037 (2)	0.044 (3)	0.039 (2)	−0.001 (2)	0.0092 (18)	0.000 (2)

### (Nitrato-κ2O,O')(1,4,7,10-tetraazacyclododecane-κ4N)nickel(II) nitrate Geometric parameters (Å, º)

**Table:**

Ni1—O1	2.151 (3)	C1—H1B	0.9700
Ni1—O3	2.113 (3)	C1—C12	1.521 (5)
Ni1—N2	2.090 (3)	C3—H3A	0.9700
Ni1—N5	2.048 (3)	C3—H3B	0.9700
Ni1—N8	2.092 (3)	C3—C4	1.515 (5)
Ni1—N11	2.065 (3)	C4—H4A	0.9700
O1—N1	1.269 (4)	C4—H4B	0.9700
O2—N1	1.221 (4)	C6—H6A	0.9700
O3—N1	1.283 (4)	C6—H6B	0.9700
N2—H2	0.9800	C6—C7	1.520 (5)
N2—C1	1.496 (5)	C7—H7A	0.9700
N2—C3	1.484 (4)	C7—H7B	0.9700
N5—H5	0.9800	C9—H9A	0.9700
N5—C4	1.481 (4)	C9—H9B	0.9700
N5—C6	1.483 (4)	C9—C10	1.516 (5)
N8—H8	0.9800	C10—H10A	0.9700
N8—C7	1.487 (4)	C10—H10B	0.9700
N8—C9	1.484 (4)	C12—H12A	0.9700
N11—H11	0.9800	C12—H12B	0.9700
N11—C10	1.471 (4)	O4—N3	1.248 (4)
N11—C12	1.467 (4)	O5—N3	1.218 (4)
C1—H1A	0.9700	O6—N3	1.218 (4)
			
O3—Ni1—O1	60.82 (10)	C12—C1—H1A	109.6
N2—Ni1—O1	98.38 (11)	C12—C1—H1B	109.6
N2—Ni1—O3	100.29 (12)	N2—C3—H3A	109.5
N2—Ni1—N8	161.91 (12)	N2—C3—H3B	109.5
N5—Ni1—O1	159.07 (12)	N2—C3—C4	110.9 (3)
N5—Ni1—O3	98.27 (11)	H3A—C3—H3B	108.1
N5—Ni1—N2	85.77 (12)	C4—C3—H3A	109.5
N5—Ni1—N8	85.96 (12)	C4—C3—H3B	109.5
N5—Ni1—N11	103.55 (12)	N5—C4—C3	107.9 (3)
N8—Ni1—O1	95.08 (11)	N5—C4—H4A	110.1
N8—Ni1—O3	96.80 (11)	N5—C4—H4B	110.1
N11—Ni1—O1	97.33 (12)	C3—C4—H4A	110.1
N11—Ni1—O3	158.13 (11)	C3—C4—H4B	110.1
N11—Ni1—N2	82.85 (12)	H4A—C4—H4B	108.4
N11—Ni1—N8	83.49 (12)	N5—C6—H6A	110.1
N1—O1—Ni1	91.2 (2)	N5—C6—H6B	110.1
N1—O3—Ni1	92.5 (2)	N5—C6—C7	108.2 (3)
O1—N1—O3	115.5 (3)	H6A—C6—H6B	108.4
O2—N1—O1	122.6 (4)	C7—C6—H6A	110.1
O2—N1—O3	121.9 (4)	C7—C6—H6B	110.1
Ni1—N2—H2	109.8	N8—C7—C6	110.1 (3)
C1—N2—Ni1	108.4 (2)	N8—C7—H7A	109.6
C1—N2—H2	109.8	N8—C7—H7B	109.6
C3—N2—Ni1	105.5 (2)	C6—C7—H7A	109.6
C3—N2—H2	109.8	C6—C7—H7B	109.6
C3—N2—C1	113.5 (3)	H7A—C7—H7B	108.2
Ni1—N5—H5	109.1	N8—C9—H9A	109.5
C4—N5—Ni1	105.7 (2)	N8—C9—H9B	109.5
C4—N5—H5	109.1	N8—C9—C10	110.5 (3)
C4—N5—C6	117.2 (3)	H9A—C9—H9B	108.1
C6—N5—Ni1	106.2 (2)	C10—C9—H9A	109.5
C6—N5—H5	109.1	C10—C9—H9B	109.5
Ni1—N8—H8	110.2	N11—C10—C9	107.7 (3)
C7—N8—Ni1	105.0 (2)	N11—C10—H10A	110.2
C7—N8—H8	110.2	N11—C10—H10B	110.2
C9—N8—Ni1	108.1 (2)	C9—C10—H10A	110.2
C9—N8—H8	110.2	C9—C10—H10B	110.2
C9—N8—C7	113.0 (3)	H10A—C10—H10B	108.5
Ni1—N11—H11	110.6	N11—C12—C1	107.5 (3)
C10—N11—Ni1	103.6 (2)	N11—C12—H12A	110.2
C10—N11—H11	110.6	N11—C12—H12B	110.2
C12—N11—Ni1	103.1 (2)	C1—C12—H12A	110.2
C12—N11—H11	110.6	C1—C12—H12B	110.2
C12—N11—C10	117.6 (3)	H12A—C12—H12B	108.5
N2—C1—H1A	109.6	O5—N3—O4	120.0 (4)
N2—C1—H1B	109.6	O5—N3—O6	120.4 (4)
N2—C1—C12	110.3 (3)	O6—N3—O4	119.6 (4)
H1A—C1—H1B	108.1		
			
Ni1—O1—N1—O2	−179.3 (3)	N2—C1—C12—N11	−50.0 (4)
Ni1—O1—N1—O3	1.0 (3)	N2—C3—C4—N5	−55.3 (4)
Ni1—O3—N1—O1	−1.0 (3)	N5—C6—C7—N8	56.3 (4)
Ni1—O3—N1—O2	179.3 (3)	N8—C9—C10—N11	52.0 (4)
Ni1—N2—C1—C12	17.8 (4)	C1—N2—C3—C4	−83.8 (4)
Ni1—N2—C3—C4	34.8 (4)	C3—N2—C1—C12	134.6 (3)
Ni1—N5—C4—C3	45.1 (3)	C4—N5—C6—C7	−160.9 (3)
Ni1—N5—C6—C7	−43.1 (3)	C6—N5—C4—C3	163.1 (3)
Ni1—N8—C7—C6	−38.1 (4)	C7—N8—C9—C10	−138.3 (3)
Ni1—N8—C9—C10	−22.5 (4)	C9—N8—C7—C6	79.5 (4)
Ni1—N11—C10—C9	−53.5 (3)	C10—N11—C12—C1	168.9 (3)
Ni1—N11—C12—C1	55.7 (3)	C12—N11—C10—C9	−166.4 (3)

### (Nitrato-κ2O,O')(1,4,7,10-tetraazacyclododecane-κ4N)nickel(II) nitrate Hydrogen-bond geometry (Å, º)

**Table:**

*D*—H···*A*	*D*—H	H···*A*	*D*···*A*	*D*—H···*A*
N2—H2···O4^i^	0.98	2.22	3.091 (5)	147
N5—H5···O3^ii^	0.98	2.32	3.130 (4)	139
N8—H8···O5^iii^	0.98	2.28	3.253 (5)	172
N8—H8···O6^iii^	0.98	2.50	3.273 (5)	135
N11—H11···O4	0.98	2.14	3.030 (5)	151

Symmetry codes: (i) −*x*+1/2, *y*+1/2, −*z*+3/2; (ii) −*x*+1, −*y*+1, −*z*+1; (iii) *x*+1, *y*, *z*.
